# Bilateral Ureteric Obstruction Due to Retroperitoneal Fibrosis: A Case Report

**DOI:** 10.7759/cureus.28187

**Published:** 2022-08-19

**Authors:** Emmanuel Okpii, Kingsley Okpii, Fatima Adamu-Biu

**Affiliations:** 1 Urology, North West Anglia NHS Foundation Trust, Peterborough, GBR; 2 Respiratory Medicine, University Hospitals of Leicester NHS Trust, Leicester, GBR

**Keywords:** uteral obstruction, bilateral ureteric stents, ormond's disease, retroperitoneal mass, retroperitoneal fibrosis

## Abstract

Retroperitoneal fibrosis is a rare disease with a largely unknown aetiology, varying presentation, and is characterized by fibrous tissue formation in the retroperitoneal region. It causes entrapment and obstruction of retroperitoneal tubular structures, notably the ureters, and has been associated with autoimmune disorders.

We report a 52-year-old male who was admitted to the emergency department with a seven-day history of lower abdominal pain, anorexia, and unintentional weight loss. Routine blood work revealed the patient to have acute kidney injury, and an unenhanced computed tomography scan of the abdomen showed bilateral hydronephrosis (grades 1 and 3 on the right and left, respectively) caused by a soft tissue mass in the retroperitoneal region. This mass was investigated with further imaging and a core biopsy, which confirmed retroperitoneal fibrosis.

He is currently being planned for ureterolysis after a poor response to steroid therapy under the nephrology team. Urinary diversion was achieved with bilateral nephrostomies following unsatisfactory drainage with bilateral ureteric stents. This case highlights some of the difficulties that may be encountered in the management of retroperitoneal fibrosis.

## Introduction

Retroperitoneal fibrosis is a rare disease characterized by the formation of fibrous tissue in the retroperitoneal region, chiefly around the anterior fourth and fifth lumbar vertebrae [1]. It is largely of unknown aetiology and can be classified as idiopathic (Ormond’s disease) or secondary, with an estimated annual incidence of one case per 200,000-500,000 population [2].

First described in 1905 by Albarran [3], it causes entrapment with consequent obstruction of tubular retroperitoneal structures, notably the ureters. It presents in a myriad of non-specific ways; thus, a high level of suspicion is required to arrive at a definitive diagnosis. Symptoms include flank or abdominal pain, malaise, fever, anorexia, and weight loss, and a definitive diagnosis is from a biopsy.

Retroperitoneal fibrosis has frequently been associated with autoimmune disorders, with a small percentage occurring in metastatic disease [1]. This has led to the belief that retroperitoneal fibrosis is immunologically mediated, and this is somewhat corroborated by its response to corticosteroids and immunosuppressive therapy.

## Case presentation

History and examination findings

A 52-year-old male who was admitted to the emergency department with a seven-day history of lower abdominal pain, anorexia, and a two-month history of left hemiscrotal swelling and pain. 

The patient was previously in a steady state of health save for the left hemiscrotal swelling, for which his general practitioner had prescribed some antibiotics on suspicion of orchitis. He reported a noticeable reduction in scrotal size and pain; however, following completion of prescribed antibiotics, he noticed a rebound in the scrotal size with worsening lower abdominal pain.

Lower abdominal pain was associated with anorexia and unintentional weight loss of about 3kg over three months. He reported normal bowel opening, albeit small volume. There was no history of fevers or vomiting. No dysuria, abnormal penile discharge, or haematuria, and he also reported no history of trauma to the abdomen.

The patient had no significant past medical history. An ex-smoker with over 20 pack years and a World Health Organization (WHO) performance status of zero. A general exam revealed a middle-aged man in no apparent distress. An abdominal exam showed a flat abdomen, soft and non-tender to palpation, and hard stools in his rectum. A urogenital exam revealed a cystic, non-tender left hemiscrotal swelling.

Investigations

Full blood count and testicular tumor markers were unremarkable; however, ultrasound of the scrotum showed left-sided hydrocele, and kidney function revealed an estimated glomerular filtration rate (eGFR) of 12ml/min and creatinine of 465 umol/L.

**Figure 1:**
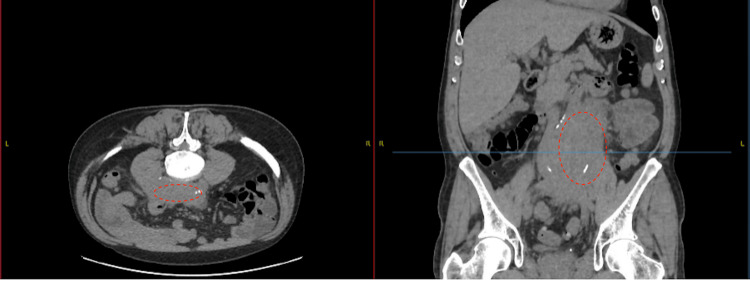
Unenhanced computed tomography scan of kidneys, ureters, and bladder Axial and coronal sections show a retroperitoneal mass in the broken red circle.

Following this finding of acute kidney injury, which wasn’t present in his previous blood tests less than a month ago, an urgent unenhanced computed tomography scan of the kidneys, ureters, and bladder (CT KUB) was performed, which showed a large lobulated soft tissue mass seen superiorly at about the level of the distal abdominal aortic bifurcation and extending inferiorly and posteriorly into the deep pelvis to below the sacroiliac joints (Figure 1). The left kidney was smaller in size and hydronephrotic (grade 3), with obstruction likely at the proximal ureter, while the right kidney was of normal size with grade 1 hydronephrosis (Figure 3). There was no evidence of left renal or ureteric stone.

These findings prompted urgent review and admission to the urology team.

**Figure 2:**
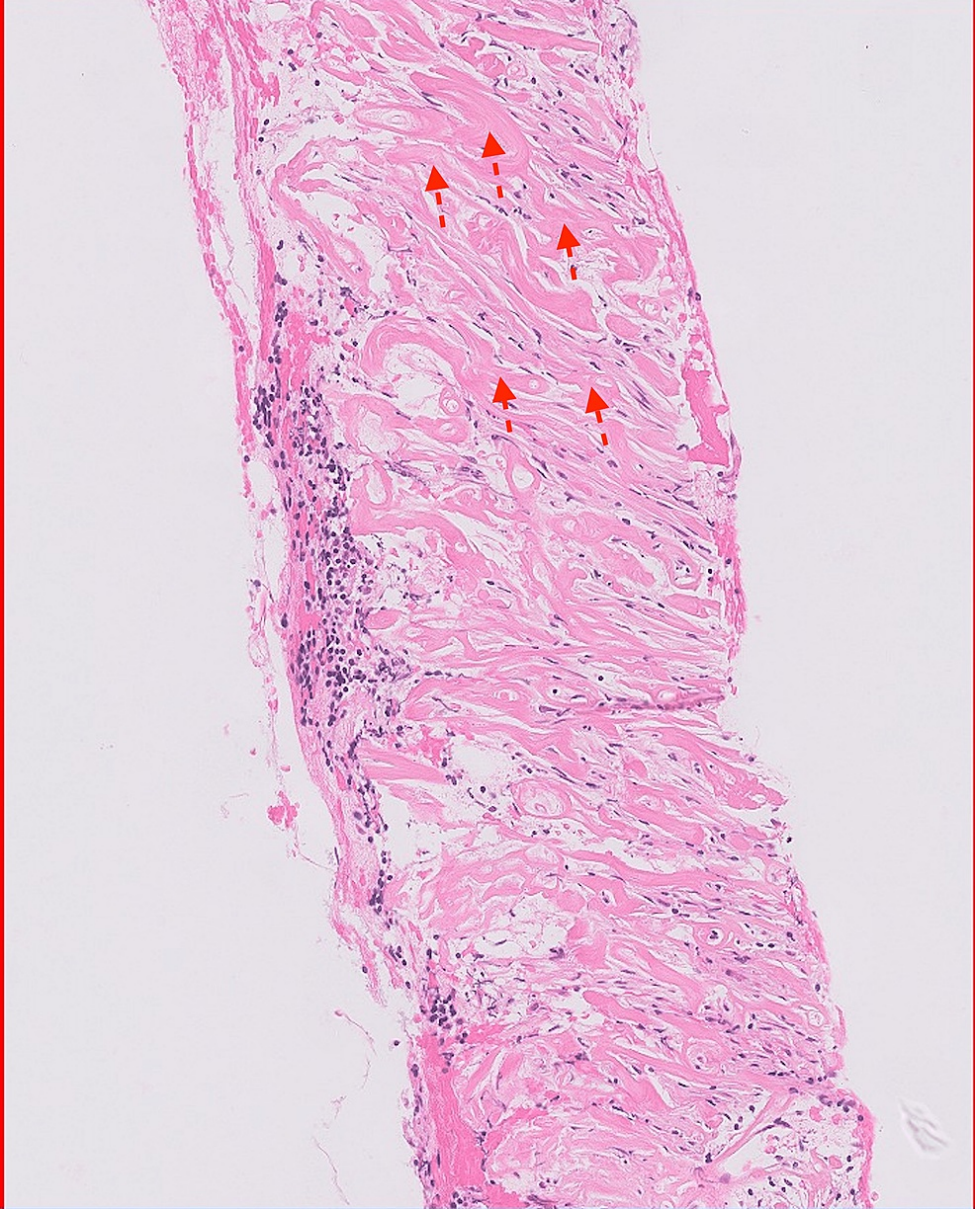
Histology slide from biopsy of index case at 100um magnification showing abundant fibrosis (broken red lines) replacing normal retroperitoneal soft tissue

Further down his care, he had a core biopsy of the retroperitoneal mass which confirmed retroperitoneal fibrosis (Figure 2). The IgG4 positive cells were non-diagnostic of IgG4 disease, and mouse double minute 2 (MDM2) amplification using fluorescent in-situ hybridization (FISH) was negative, thus excluding possible sarcoma.

Treatment

The patient was reviewed in the emergency department and informed of the investigation findings. This showed acute kidney injury with significant left-sided hydronephrosis (grade 3) due to obstruction from a retroperitoneal mass. He consented and underwent an urgent left ureteric double J stent insertion. Intra-operatively he was noted to have a very tight left ureter with a distended duplex collecting system. Multiple attempts at cannulating the ureter were unsuccessful with a 6.26Fr ureteric catheter; however, the superior duplex moiety was successfully cannulated with a 4.8Fr multi-length double J stent.

**Figure 3:**
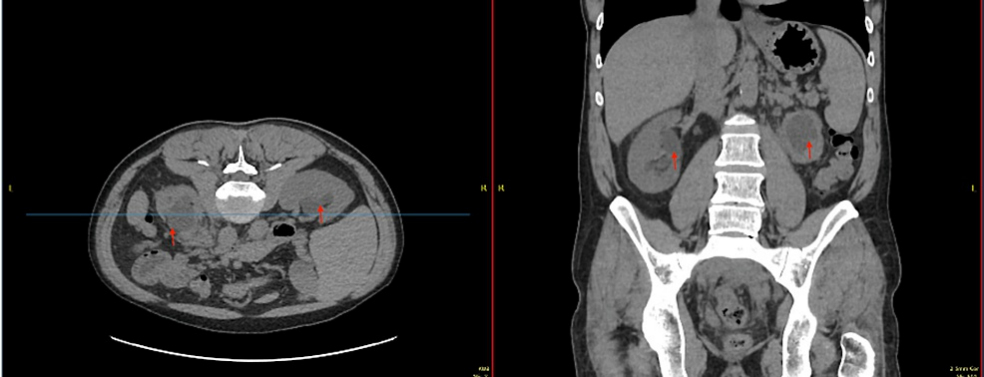
Unenhanced computed tomography scan of kidneys, ureters, and bladder Axial and coronal sections show bilateral hydronephrosis (indicated with red arrows), worse on the left.

The patient was discussed at the urology multi-disciplinary team (MDT) meeting on account of his poor kidney function despite left ureteric stent in situ and admitting scan findings. A review of previous unenhanced computed tomography scans of kidneys, ureters, and bladder showed bilateral hydronephrosis (Figure 3), and the recommendation was for bilateral ureteric double J stent insertion, and subsequent computed tomography (CT) guided biopsy of the retroperitoneal mass in the future; his kidney function had not improved sufficiently to arrange a contrast-enhanced computed tomography scan. 

Post MDT recommendation, the patient consented to a right ureteric stent insertion and left ureteric stent exchange. Intra-operative findings showed a right duplex collecting system as well, and 6.26Fr ureteric double J stents were inserted bilaterally. 

**Figure 4:**
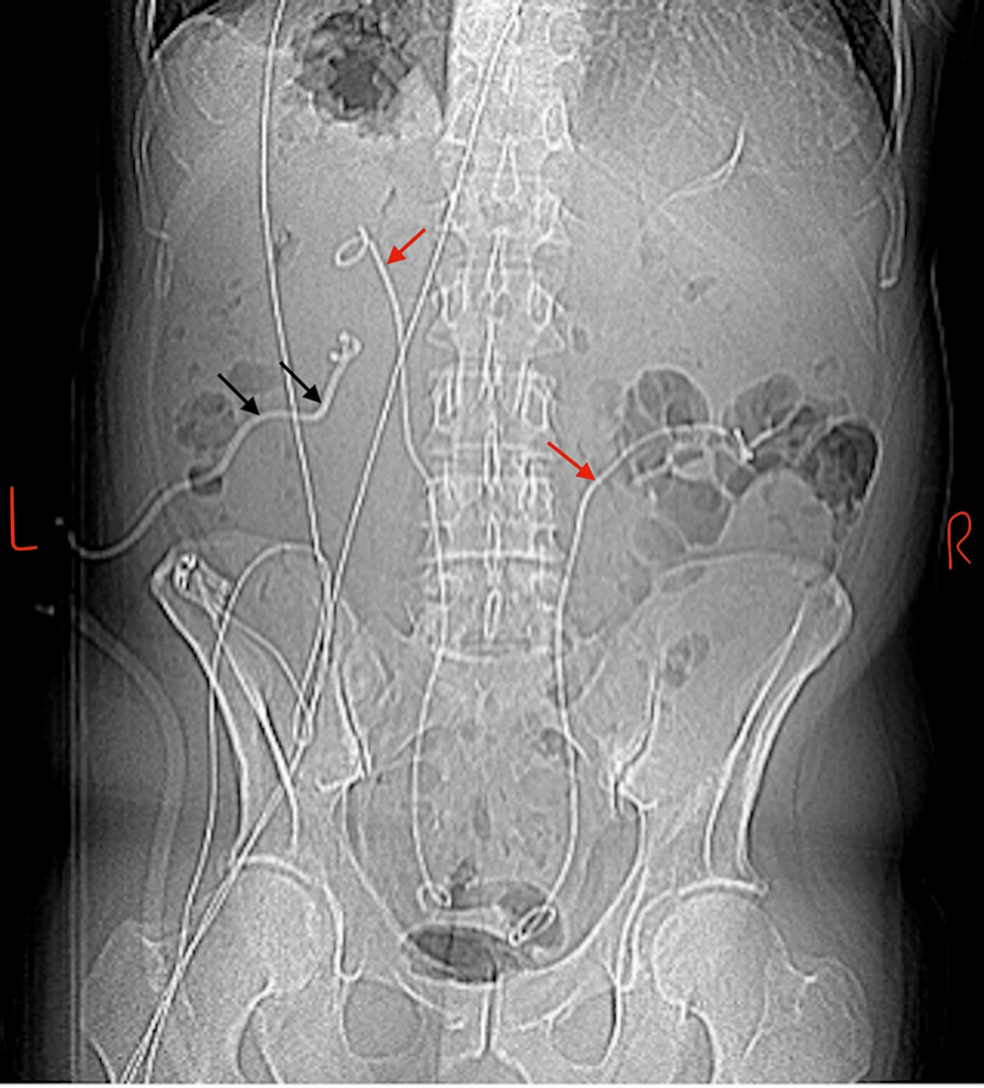
Computed tomography scan Scout image showing bilateral ureteric stents (in red arrows) and left nephrostomy (in black arrows).

Post insertion of bilateral ureteric stents, the patient’s kidney function was not improving significantly, and an ultrasound scan reaffirmed the presence of bilateral hydronephrosis, grades 1 and 3 on the right and left kidneys, respectively. He subsequently had a left nephrostomy inserted (Figure 4) which led to some improvement in his kidney function.

He was discharged home and scheduled for a re-discussion at our MDT meeting, following which he would be informed of the outcome. The outcome of the discussion was for referral to a tertiary center for a CT-guided biopsy; however, he was soon hospitalized on account of worsening dyselectrolytemia (severe hyperkalaemia, increasing creatinine) and poor urine output, which required continuous venovenous haemofiltration (CVVH) in the intensive care unit. He improved with CVVH and was transferred to a tertiary center, where he had a right nephrostomy inserted, and a biopsy of his retroperitoneal mass was done. The biopsy confirmed idiopathic retroperitoneal fibrosis (Figure 2) with IgG4 levels in the non-diagnostic range and negative FISH for MDM2 amplification. The patient was subsequently discharged with bilateral nephrostomies and ureteric stents in situ.

Outcome and follow-up

The patient remains well and reports normal appetite and energy levels. Nonetheless, he is struggling with the care of the bilateral nephrostomies and is keen to get rid of them. Comparatively, the right nephrostomy has always drained more than the left and currently produces about 100mls/hr of urine on average, while the left produces about 200mls/24hrs. His eGFR seems to be plateauing around just over 30mls/min, which, albeit a significant improvement from his first presentation, leaves him rather disappointed as he had hoped for a return to pre-admission levels. A discussion of his biopsy result was had, which reassuringly did not show any IgG4-related disease, even though the levels were slightly above the normal range.

He is planned for an interval removal of the nephrostomy catheters whilst retaining bilateral ureteric stents to improve his quality of life, and these ureteric stents will undergo routine exchange when due. He has also undergone steroid therapy but evidence has suggested poor response, and thus he is planned for ureterolysis.

## Discussion

Retroperitoneal fibrosis is an uncommon disease condition with an estimated annual incidence of one per 200,000-500,000 population [2]. Its aetiology is largely unknown; however, it has been associated with some autoimmune conditions as well as metastatic malignancy. First reported in 1905 by Albarran, it was further described by Ormond in 1948 and features fibrous tissue formation in the retroperitoneal region with frequent encasement and obstruction of structures that lay therein [1]. 

It presents a unique diagnostic challenge and this is due to a handful of reasons. Firstly, its rarity puts it on the tail end of differentials; secondly, its clinical presentation is non-specific, thirdly, many cases occur as incidental findings on imaging and lastly, confirmation is invasive, requiring biopsy.

The clinical presentation of retroperitoneal fibrosis ranges from flank or abdominal pain to constitutional symptoms like anorexia and weight loss, all of which the index patient presented with. It has also been described to coexist with other pathologies such as hydrocele [4], which the index patient had, and a similar case of retroperitoneal fibrosis seen with left-sided hydrocele was reported in 2008 in Kenya [4]. The index patient presented with non-specific clinical features, and only when investigation findings revealed him to be in acute kidney injury did a search for its cause reveal a posterior abdominal wall mass.

Management of patients with retroperitoneal fibrosis is a multi-pronged endeavor, which is geared at relieving symptoms, preserving renal function, and exclusion of secondary causes, including malignancy. Treatment is dependent on the stage of disease at diagnosis, and could be medical or surgical. Because the common clinical presentation would include abdominal or flank pain, and often kidney injury from ureteric obstruction (obstructive uropathy), interventions targeted at these are often pursued early during treatment. Ureteric obstruction can be amenable to both medical and surgical therapy; however, the latter often results in quicker resolution of symptoms. Surgical therapy could be in the form of temporizing maneuvers such as percutaneous nephrostomy or ureteric stent insertion (Figure 4), both of which the patient had; however, primary surgical management involves biopsy to confirm disease as well as ureterolysis and lateral/intraperitoneal transposition or omental wrapping of the involved ureter [5,6]. Urinary tract de-obstruction using ureteric double J stents was attempted, but this failed to achieve adequate urinary drainage with no significant improvement in the patient’s renal function, thus necessitating percutaneous nephrostomy insertion. It is noteworthy that the failure of the former was because of bilateral duplex collecting systems, which were difficult to cannulate intra-operatively, and albeit successful, adequate drainage was not achieved.

There is no consensus on medical therapy for retroperitoneal fibrosis as there is a lack of controlled therapeutic trials to reach that consensus; however, steroids and other immuno-modulators are used [1]. Following successful urinary tract de-obstruction, the patient has been commenced on steroid therapy with regular follow-up by both the urology and nephrology teams. Most recently, his follow-up has shown a poor response to steroid therapy which has warranted a further action plan involving planned ureterolysis.

Learning points

The justification for this paper hinged on the rarity of retroperitoneal fibrosis; however, we also sought to highlight some of the difficulties that may ensue in its management. To achieve adequate treatment of such a case, one must first diagnose the patient; however, due to its non-specific clinical presentation, there’s usually a low index of suspicion, which may lead to a delay in its diagnosis. Finally, we would like to highlight the different approaches in the management of retroperitoneal fibrosis, which include both medical and surgical interventions, as demonstrated in this paper.

## Conclusions

Managing retroperitoneal fibrosis can be very challenging for all parties involved, patients and clinicians alike. As has been demonstrated in the index case, it requires a multi-facet approach, including medical, surgical, and other interventional measures which may not be locally available. This may necessitate multiple visits to different hospitals with the attendant consequences therefrom. 
